# Gene-to-metabolite network for biosynthesis of lignans in MeJA-elicited *Isatis indigotica* hairy root cultures

**DOI:** 10.3389/fpls.2015.00952

**Published:** 2015-11-03

**Authors:** Ruibing Chen, Qing Li, Hexin Tan, Junfeng Chen, Ying Xiao, Ruifang Ma, Shouhong Gao, Philipp Zerbe, Wansheng Chen, Lei Zhang

**Affiliations:** ^1^Department of Pharmaceutical Botany, School of Pharmacy, Second Military Medical UniversityShanghai, China; ^2^Department of Pharmacy, Shanghai Changzheng Hospital, Second Military Medical UniversityShanghai, China; ^3^School of Traditional Chinese Materia Medica, Shenyang Pharmaceutical UniversityShenyang, China; ^4^Department of Plant Biology, University of California, DavisDavis, CA, USA

**Keywords:** *Isatis indigotica*, AP2/ERF, biosynthesis of lignans, gene-metabolic network, metabolic engineering

## Abstract

Root and leaf tissue of *Isatis indigotica* shows notable anti-viral efficacy, and are widely used as “Banlangen” and “Daqingye” in traditional Chinese medicine. The plants' pharmacological activity is attributed to phenylpropanoids, especially a group of lignan metabolites. However, the biosynthesis of lignans in *I. indigotica* remains opaque. This study describes the discovery and analysis of biosynthetic genes and AP2/ERF-type transcription factors involved in lignan biosynthesis in *I. indigotica*. MeJA treatment revealed differential expression of three genes involved in phenylpropanoid backbone biosynthesis (*IiPAL, IiC4H, Ii4CL*), five genes involved in lignan biosynthesis (*IiCAD, IiC3H, IiCCR, IiDIR*, and *IiPLR*), and 112 putative AP2/ERF transcription factors. In addition, four intermediates of lariciresinol biosynthesis were found to be induced. Based on these results, a canonical correlation analysis using Pearson's correlation coefficient was performed to construct gene-to-metabolite networks and identify putative key genes and rate-limiting reactions in lignan biosynthesis. Over-expression of *IiC3H*, identified as a key pathway gene, was used for metabolic engineering of *I. indigotica* hairy roots, and resulted in an increase in lariciresinol production. These findings illustrate the utility of canonical correlation analysis for the discovery and metabolic engineering of key metabolic genes in plants.

## Introduction

**Figure 1:**
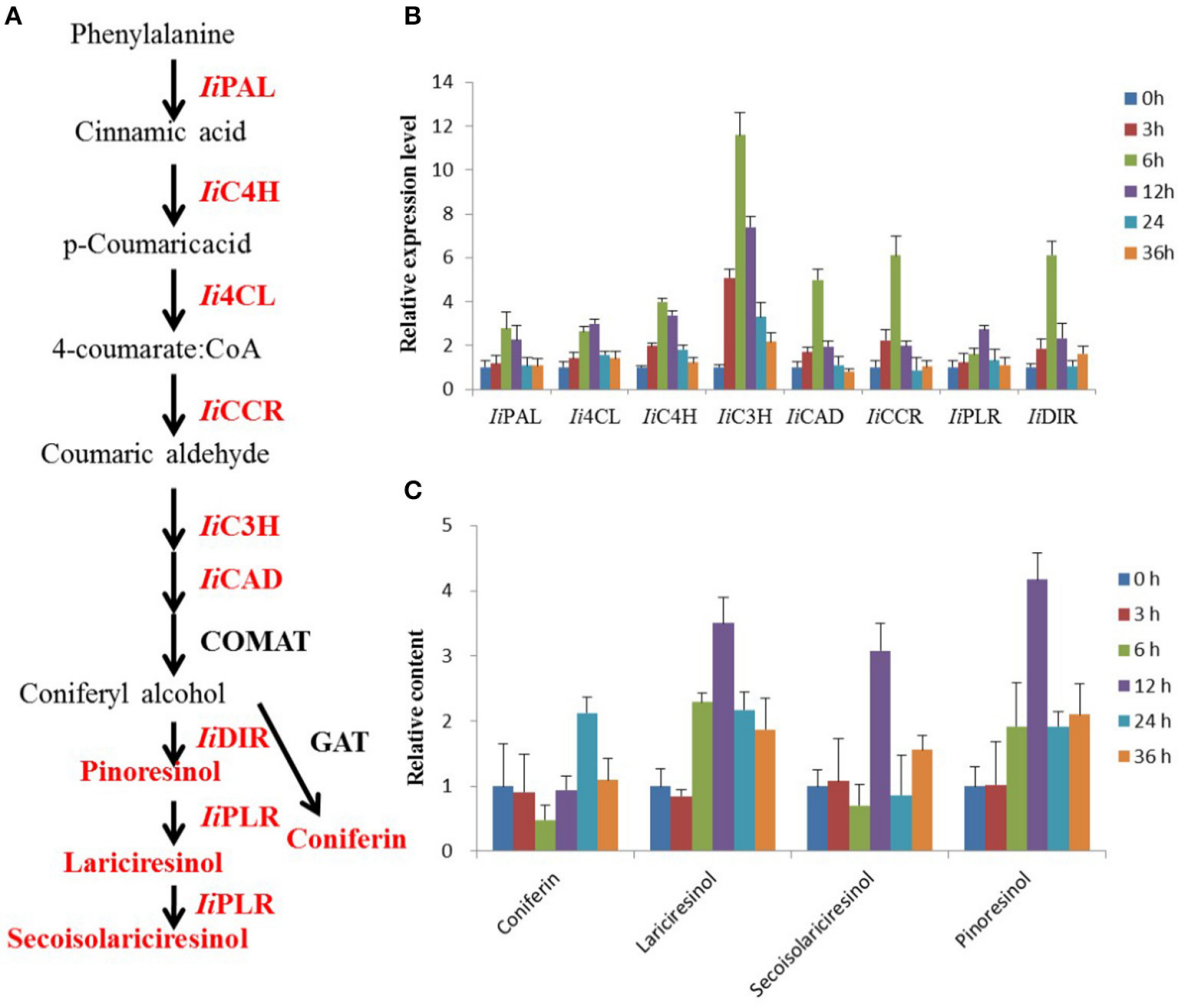
**MeJA-induced changes in lignan biosynthesis analyzed via transcript and metabolite profiling**. **(A)** Schematic illustration of the lignan biosynthetic pathway in *I. indigotica*. Red colored proteins and metabolites were analyzed in this study. **(B)** Effect of MeJA treatment on transcript abundance of biosynthetic genes, *n* = 3. Effect of MeJA treatment on accumulation of four key metabolites in lignan biosynthesis, *n* = 3.

*Isatis indigotica* Fortune has been used in traditional Chinese medicine for more than two millennia and is listed in the Chinese Pharmacopoeia (National Pharmacopoeia Committee, 2010). The root and leaves of *I. indigotica* demonstrate notable anti-viral (Chang et al., 2012), anti-inflammatory (Tang et al., 2014), anti-tumor (Chung et al., 2011), and anti-anaphylaxis (Recio et al., 2006) activity, and are used in clinical applications as “Banlengen” and “Daqingye,” respectively. In previous researches, lignans including lariciresinol and larch lignan glycosides were considered as the material base of those activities (Yang et al., 2013). However, the biosynthesis of lignans in *I. indigotica* is largely unresolved. Transcriptome analysis of *I. indigotica* (Chen et al., 2013) and availability of the complete genomes of other lignan-forming plant species (*A. thaliana* and Chinese cabbage) offer the opportunity to employ bioinformatics tools for better understanding and ultimately modulating lignan metabolism in *I. indigotica*. In addition, key genes responsible for the biosynthesis of backbone structures of phenylpropanoids, flavonoids, lignans and lignins have been established, including phenylalanine-ammonia lyase (PAL), cinnamate-4-hydroxylase (C4H) and coumaroyl-CoA-ligase (4CL) of phenylpropanoid metabolism, chalcone synthase (CHS), flavonol synthase (FNS) and chalcone isomerase (CHI) of flavonoid biosynthesis, and cinnamoyl alcohol dehydrogenase (CAD) and cinnamoyl-CoA reductase (CCR) in lignan formation (Figure 1A).

TFs are essential for the coordination of metabolic pathways involved in plant development and environmental stress responses to, for example, drought, salt stress, high temperature, and other abiotic perturbations (Li et al., 2014a,b; Tavakol et al., 2014). Containing at least one AP2 DNA-binding domain, AP2/ERF transcription factors form an important TF superfamily with roles in biotic and abiotic stress responses (Filiz and Tombuloğlu, 2014; Lee et al., 2014). AP2/ERF TFs are divided into four families, ERF, AP2, RAV, and Soloist (Thamilarasan et al., 2014). The ERF family further comprises two subfamilies, ERF and DREB. AP2 and RAV contain two domains, comprised of two AP2 domains in members of the AP2 family, while members of the RAV family contain one AP2 domain and one B3 domain (Song et al., 2013; Sun et al., 2014). Despite a high sequence identity, the members AP2/ERF family show a large diversity regarding their DNA-binding motifs (Qin et al., 2007; Hong et al., 2009; Wang et al., 2011) and functions (Hong and Kim, 2005; Ito et al., 2006; Fujita et al., 2011; Table 1).

**Table 1:** **The DNA-binding sequences and main functions of AP2/ERF superfamily**.

**Family name**	**DNA-binding**	**Main function**	**Examples**
DREB subfamily	CCGAC	Enhance abiotic stress tolerance	*AtCBF1* (low temperature)
			*ZmDREB2A* (heat)
			*OsDREB1* (drought)
			*CaDREBLP1* (high salt)
ERF subfamily	AGCCGCC	Direct plant defense to abiotic and biotic stress	PR1 to PR5 genes (pathogen)
			AP37 (salt)
			SNORKEL1 (hypoxic stress)
AP2 family	GCAC (A/G) N (A/T) TCCC (A/G) ANG (C/T)	Plant development	NsAP2 (plant height and leaf shape)
RAV family	CAACA and CACCTG	Mediate plant defense to abiotic and biotic stress	CaRAV1 (high salt and osmotic stress)
Soloist family	–	Defense against bacterial pathogens	At4g13040 (Ethylene-responsive)

**Figure 2:**
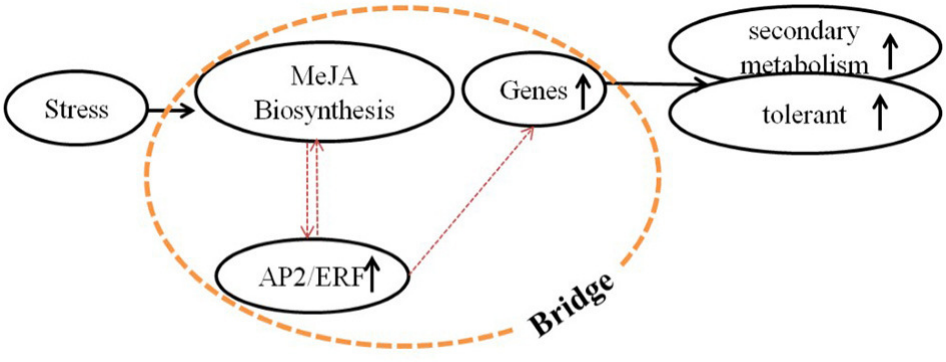
**Molecular mechanism of regulation by signal molecules**. Molecular mechanism of regulation among environmental stress, signal molecules, and secondary metabolism. A bridge that consists of MeJA biosynthesis (or other signal pathways), AP2/ERFs (or other TFs) and biosynthetic genes was supposed, which connected the environment and secondary metabolism. This bridge was mainly studied in this paper.

Essential roles for AP2/ERF TFs (AP2/ERFs) in the response to abiotic (drought, salt, and temperature) and biotic stress factors have been demonstrated for numerous plant species, such as rice, tobacco, and tomato (Pan et al., 2012; Zhang et al., 2013, 2014; Wu et al., 2014). In addition, some AP2/ERFs, ORA47 (Pauwels et al., 2008) in *Arabidopsis thaliana* and RAV1 (Himi et al., 2011) in wheat, were reported to have the possibility of interaction with genes of biosynthesis of lignins and flavonoids. However, a role of AP2/ERFs in lignan biosynthesis has so far not been investigated. Conversely, the role of phytohormones, including methyl jasmonate (MeJA) (Yan et al., 2014), salicylic acid (SA) (D'Maris et al., 2011), and abscisic acid (ABA) (Finkelstein, 2013), in the regulation of phenylpropanoid biosynthetic pathways has been established (Agrawal et al., 2014; Liu et al., 2014). We therefore assume that a “bridge” consisting of AP2/ERFs, phytohormones and biosynthetic genes, connects the environment stresses and phenylpropanoids metabolism (Figure 2) (Pré et al., 2008; Zhang et al., 2012).

In this study, 112 putative AP2/ERFs were identified in *I. indigotica* and analyzed using a bioinformatics approach. This included the analysis of physicochemical properties of individual AP2/ERFs, phylogenetic studies comparing AP2/ERF orthologs of *I. indigotica, A. thaliana*, and *B. rapa*. Transcript profiling revealed differential expression patterns of select AP2/ERF candidates. In addition, key genes (biosynthetic genes and AP2/ERFs) observed to significantly impact lignan biosynthesis were identified by correlating transcript and metabolite analyses of MeJA-treated tissues. These results enabled the selection of high-probability genes, and the downstream metabolic engineering of lignan biosynthesis in *I. indigotica* hairy roots. Here, over-expression of *Ii*C3H increased lariciresinol production by 4.5-fold.

## Materials and methods

### Plant material

Plants of *I. indigotica* were grown at university greenhouses (Second Military Medical University, Shanghai, China,). Species verification was performed by Professor Hanming Zhang of the School of Pharmacy (Second Military Medical University).

The sterile *I. indigotica* plants were grown and kept in our greenhouse. The sterile leaf sections were submerged in the bacterial suspension for 30 min to induce hairy roots of *I. indigotica*, which were then placed on MS medium supplemented with 30% sucrose, 0.8% agar (pH 5.8), at 25°C and under dark conditions. Cultures were then washed three times with 60 mL sterilized water, blot-dried on sterile filter paper, and transferred to ½ MS medium (as above) and supplemented with 500 mg·L^−1^ cefotaxime after 3 days. After 3 weeks, hairy roots were isolated from leaves and cultivated for 3–4 weeks (25°C, darkness) on solid ½ MS medium (as above) with successive subcultures being grown on decreasing cefotaxime concentrations (250, 100, 0 mg·L^−1^). Rapidly growing root cultures lacking bacterial contamination were further used to establish hairy root lines. Approximately 200 mg of normally growing hairy roots were inoculated in 200 mL ½ MS liquid medium and grown in 250 mL shaking flasks at 100 rpm, 25°C and darkness. Clonal hairy root cultures were routinely subcultured every 30 days, treated by MeJA and harvested after 60 days.

Treatments were designated: (1) 0.5 μM of MeJA (Sigma, USA) dissolved in ethanol was added to 200 mL of 1/2 MS liquid medium; (2) Ethanol at the same volume was added into the control group. After treatment, the plants were harvested at 0, 1, 3, 6, 12, 24, and 36 h. Three independent biological replicates for each group.

### Identification of AP2/ERFs

For the identification of candidate AP2/ERF genes, a previously established *I. indigotica* transcriptome inventory was used (Chen et al., 2013). The assembled transcriptome was queried against 159 known *A. thaliana* AP2/ERF proteins (*At*AP2/ERF) retrieved from the Database of *Arabidopsis* Transcription Factors (DATF, http://datf.cbi.pku.edu.cn/) and 321 Chinese cabbage AP2/ERF proteins (*Bra*AP2/ERF) obtained from the *Brassica* Database (BRAD, http://brassicadb.org/brad/) to select AP2/ERF gene candidates (TBLASTN with a *E*-value cut-off of 10^−5^). After removing sequences with bit scores less than 100 or alignment length less than 100 bp, the left sequences were screened in the Pfam database (pfam, http://pfam.janelia.org/) to identify the AP2/ERF proteins with default parameters. Finally, as a quality check, using the Simple Modular Architecture Research Tool (SMART, http://smart.embl-heidelberg.de/).

### Sequence analysis

The full-length ORF sequences of the 112 putative AP2/ERFs were obtained and converted into amino acid sequences by Vector NTI Advance (TM) 11.5 and MEGA 5.05. Using the ProtParam tool (http://web.expasy.org/protparam). Secondary structure of AP2/ERFs were predicted using the Secondary Structure Prediction Method (SOPMA, http://npsa-pbil.ibcp.fr/cgi-bin/npsa_automat.pl?page=/NPSA/npsa_sopma.html). ClustalX 2 was used to accurately identify AP2/ERF domains. Conserved amino acid motifs were identified using Multiple EM for Motif Elicitation (MEME, http://meme.nbcr.net/meme/cgi-bin/meme.cgi) with default settings. *Ii*C3H and other *C3H*s obtained from Genbank were aligned and a Neighbor-Joining (NJ) tree was constructed by MAGA 5.05 (http://www.megasoftware.net/).

### Transcript abundance of AP2/ERFs in *I. indigotica* hairy roots treated with MeJA

To get insight into the AP2/ERFs' transcript abundance induced with MeJA in *I. indigotica*, the Illumia RNA-Seq data in previous research was utilized (Chen et al., 2013). The RNA-Seq expression profile data were generated using the Illumia HiSeq™ 2000 platform, and included the hairy roots of *I. indigotica* treated with MeJA at 0, 1, 3, 6, 12, and 24 h. Zero hour was used as control to normalized expression level data in MultiExperiment Viewer (Saeed et al., 2003).

### Phylogenetic analysis of AP2/ERFs

The amino acid sequence alignments of AP2/ERF proteins were performed by Clustal W. NJ method with pairwise deletion option in MEGA 5.05 was used to analyze the phylogenetic and molecular evolutionary genetics. Reliability of the tree was estimated using a bootstrap analysis with 1000 replicates. Based on the original dataset, bootstrap values above 50% were added to the tree branches. The AP2/ERFs were searched for duplication events (*e* < le–10, identity > 90%) in *I. indigotica*.

### Quantitative real-time PCR

High quality total RNA (1 μg) was used to prepare first-strand cDNA using the TransScript First-Strand cDNA Synthesis SuperMix kit (TransGen Biotech, Beijing, China) following the manufacturer's protocol.

Quantitative real-time PCR (qRT-PCR) was performed according to the manufacturer's instructions using a TP8000 Real-time PCR detection system and the SYBR premix Ex Taq kit (TAKARA, Japan) with the following PCR program: 95°C for 30 s, followed by 40 cycles of 95°C for 5 s, 53°C for 10 s, and 72°C for 20 s. All PCR reactions consisted of three technical replicates. Transcript abundance of each gene was normalized to *ubiquitin* with the comparative C_t_ method (Livak and Schmittgen, 2001; Udvardi et al., 2008). Oligonucleotides used in this study are given in Table S1. Three independent biological replicates for each sample and three technical replicates for each biological replicate were analyzed.

### Metabolites analysis

Dried hairy roots (50 mg) were ground into a fine powder and extracted twice with 25 mL of 80% methanol under sonication for 30 min. After centrifugation, the supernatant was diluted with 80% methanol to a total volume of 50 mL, and filtered through a 0.22 μm organic membrane filter prior to HPLC analysis. HPLC analysis was conducted on an Agilent 1200 series instrument with an Agilent 6410 triple-quadrupole mass spectrometer and an electrospray ionization source (Agilent Corporation, MA, USA). Metabolite separation was achieved on an Agilent ZORBAX SB-C18 column (3.5 μm, 2.1 × 150 mm) and an Agilent C18 guard column (5 μm, 4.0 × 2.0 mm). The mobile phase was acetonitrile: 5 mM ammonium acetate solution (the concentration of acetonitrile was from 5 to 95% in 1.0 min, v/v) with the flow rate of 0.3 mL·min^−1^ and a total run time of 5 min. Metabolite identification and quantification was achieved in multiple reaction monitoring mode (MRM). Characteristic *m*/*z* ions are listed in Table S2. The samples for qRT-PCR and metabolites analysis were the same.

### Integration of transcript and metabolite analyses

Correlation analysis integrating transcript and metabolite data of control and MeJA-induced hairy root cultures was performed by canonical correlation analysis using Pearson's correlation coefficient (Xiao et al., 2009). Gene-to-metabolite, TF-to-gene and TF-to-metabolite networks were visualized to identify probable key genes in lignan biosynthesis.

### Plant transformation and growth of hairy root culture

The full-length *Ii*C3H was inserted into vector pCAMBIA1304 to obtain pCAMBIA1304-*Ii*C3H. Sterile *I. indigotica* plants were grown and kept in our greenhouse. The disarmed *A. tumefaciens* strain C58C1 harboring both the *A. rhizogenes* Ri plasmid pRiA4 (Kai et al., 2009) and plasmid constructed above was used for plant genetic transformation.

The method of growth of transgenic hairy root culture was similar to process in 2.1. However, hygromycin (10 mg·L^−1^) should be added with cefotaxime. Rapidly growing root cultures showing hygromycin resistance and lacking bacterial contamination were further used to establish hairy root lines. Approximately 200 mg of normally growing hairy roots were inoculated in 200 mL ½ MS liquid medium and grown in 250 mL shaking flasks at 100 rpm, 25°C and darkness. Clonal hairy root cultures were routinely subcultured every 30 days and harvested after 60 days.

### PCR analysis of hairy root culture

Genomic DNA was isolated from hairy root samples using the acetyl trimethyl ammonium bromide (CTAB) method (Doyle and Doyle, 1990). Then the DNA was used in PCR analysis for detecting the presence of the specific genes in transgenic lines. Primer sequences for amplifying these genes (these primers were particularly designed to cover the gene sequence and the vector sequence for detecting exogenous gene transformations) are listed in Table S1. The selectable marker hygromycin resistance gene *hph* was used to check the pCAMBIA1304 vector transformants, whereas *Agrobacterium* gene *rolb* and *rolc* were used to check the transformation of pRiA4 (Chilton et al., 1982). The PCR reaction program: 94°*C* for 3 min followed by 35 cycles of amplification (94°*C* for 10 s, 58°*C* for 30 s, 72°*C* for 1 min) with final extension at 72°*C* for 5 min.

### Statistical analysis

Statistical analysis was performed with SPSS 13.0 software. Analysis of variance (ANOVA) was followed by Tukey's pairwise comparison tests, at a level of *p* < 0.01, to determine significant differences between means.

## Results

### Analysis of AP2/ERFs in *I. indigotica*

#### Identification of AP2/ERFs in the *I. indigotica* transcriptome

A total of 112 putative AP2/ERFs, designated *Ii*001 to *Ii*112, were obtained through query of a previously established *I. indigotica* transcriptome inventory (Chen et al., 2013) against public AP2/ERF and AP2/ERFs-like protein sequences of *A. thaliana* and *B. rapa* by TBLASTN (Basic Local Alignment Search Tool 2.2.26) (Table S3). The best hit homology genes of these sequences to *A. thaliana* and *B. rapa* were summarized (Table S3) and the AP2/ERF proteins were subsequently categorized by domain types (http://pfam.sanger.ac.uk/). A total of 42 ERF, 45 DREB, 20 AP2, 3 RAV, and 2 Soloist gene candidates were identified, all of which contained characteristic domain features (SMART, http://smart.embl-heidelberg.de/) (Table S3).

#### Sequence analysis

Sequence analysis of the 112 identified AP2/ERF demonstrated ORF lengths ranging from 92 aa (*Ii*015) to 565 aa (*Ii*037) and the molecular masses varied from ~10.29 (*Ii*015) to 625.11 kDa (*Ii*037) (Table S4). This differences are, in part, resulting from incomplete sequencing. The predicted *p*I values ranged from 4.42 (*Ii*036) to 11.58 (*Ii*069) and instability indices varied between 23.98 (*Ii*055) and 81.28 (*Ii*015) with an average value of 54.66. Aliphatic indices ranged from 44.33 (*Ii*111) to 82.48 (*Ii*069) averaging at 62.54, and hydrophobicity values of all the AP2/ERF proteins were below zero, ranging from -0.079 (*Ii*112) to -1.17 (*Ii*097). Secondary structure prediction indicated a predominantly random coils (53.28%), with α-helical folding pattern (28.02%), extended strands (13.68%) and β-turns (5.01%) (Table S5).

Computational prediction of the subcellular localization (WoLF PSORT; http://www.genscript.com/psort/wolf_psort.html) placed the majority of the identified AP2/ERFs at the nucleus with a few gene candidates showing a possible localization in mitochondria, Golgi apparatus, cytoplasm and chloroplasts (Table S6). With the exception of *Ii*095 (for which a 19 aa N-terminal transit peptide was predicted), no signal peptides were observed in the identified AP2/ERF candidates using the NetNGlyc 1.0 server (http://www.cbs.dtu.dk/services/NetNGlyc/).

#### Phylogenetic analysis of the *I. indigotica* AP2/ERF superfamily

**Figure 3:**
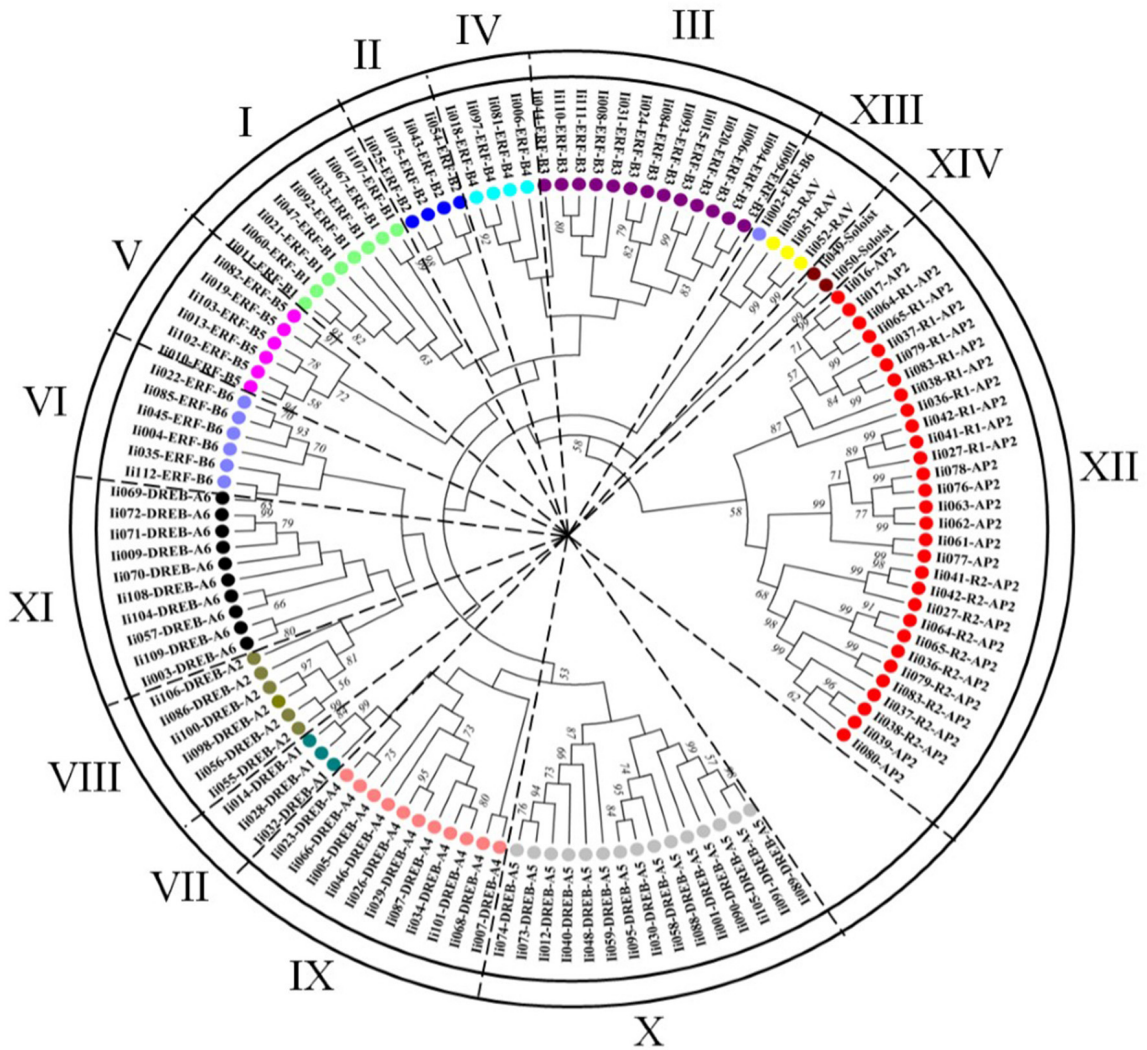
**Phylogenetic analysis of the *I. indigotica* AP2/ERFs family**. The phylogenetic tree of AP2/ERF transcription factor domains identified in *I. indigotica* was constructed using the neighbor-joining approach with 1000 bootstrap repetitions. Based on the topology of the generated phylogenetic tree it can be inferred that the AP2/ERFs family of *I. indigotica* is divided into 14 groups (I–XIV, without DREB-A3), containing the ERF (DREB and ERF subfamilies), AP2, RAV, and Soloist families. Different subfamilies are color coded.

To gain a detailed understanding of evolutionary interrelations and the topological structure of the *I. indigotica* AP2/ERF protein family a neighbor-joining phylogenetic tree was constructed (Figure 3), which contained the DREB and ERF subfamilies, and the AP2, RAV and Soloist families that were further divided into 14 clades (without DREB-A3). Groups I–VI represented the ERF subfamily, groups VII–XI the DREB subfamily, and groups XII, XIII, and XIV comprised the AP2, RAV, and Soloist families, respectively. The DREB subfamily comprised the largest number of members, followed by the ERF, AP2, RAV, and Soloist families.

Duplication events had already been learned in grape and Chinese cabbage. Seventeen and fifteen proteins with sequences of a high similarity were reported, respectively (>95% sequence similarity) (Song et al., 2013). Similarly, this study identified 19 presumably duplicated genes in *I. indigotica* sharing 95% sequence similarity. Among these genes, 11 were classified as DREB subfamily genes, while the remaining eight genes were annotated as AP2 proteins (Table 2).

**Table 2:** **Gene duplication of AP2/ERF superfamily in *I. indigotica***.

***Ii-*AP2 (ID1)**	***Ii*-AP2 (ID2)**	**Identity (%)**
*Ii*041-AP2	*Ii*061-AP2	100
*Ii*042-AP2	*Ii*061-AP2	100
*Ii*027-AP2	*Ii*061-AP2	97.62
*Ii*061-AP2	*Ii*042-AP2	95.35
*Ii*077-AP2	*Ii*027-AP2	97.73
*Ii*077-AP2	*Ii*041-AP2	97.73
*Ii*076-AP2	*Ii*077-AP2	98.41
*Ii*077-AP2	*Ii*078-AP2	98.41
*Ii*069-DREB-A6	*Ii*070-DREB-A6	100
*Ii*076-AP2	*Ii*078-AP2	100
*Ii*061-AP2	*Ii*062-AP2	100
*Ii*061-AP2	*Ii*063-AP2	100
*Ii*073-DREB-A5	*Ii*074-DREB-A5	98.88
*Ii*042-AP2	*Ii*041-AP2	100
*Ii*062-AP2	*Ii*063-AP2	99.62
*Ii*055-DREB-A2	*Ii*056-DREB-A2	100
*Ii*038-AP2	*Ii*039-AP2	100
*Ii*016-AP2	*Ii*017-AP2	98.88

To comprehensively analyze the evolutionary diversification of the *I. indigotica* AP2/ERF superfamily an additional phylogenetic tree was generated that compared all 112 identified AP2/ERF proteins of *I. indigotica*, 289 proteins of *B. rapa*, and 148 proteins of *A. thaliana*, inclusive of the DREB and ERF subfamilies, as well as AP2, RAV, and Soloist families that were further divided into 15 subgroups (Figure S1). The generated tree illustrated the ERF family (ERF and DREB subfamilies) and the Soloist family as the largest and smallest clusters, respectively. Notably, the ERF subfamily comprised two separate subgroups, which, in turn were divided into six (B1–B6) and two (B1 and B6) clusters, respectively. This result may indicate a more expansive evolutionary divergence of the B1–B6 groups.

To further clarify the relationships among AP2/ERF proteins in *I. indigotica*, multiple alignment analyses of characteristic AP2/ERF domains were performed for every subfamily. Overall, all proteins showed high sequence similarity and distinct family-specific domain features. All members of DREB subfamily and most ERF proteins contained a WLG element. In addition, the majority of DREB proteins harbored an EIR element. Most AP2 proteins contained two AP2 domains, with exception of 10 proteins that lacked the second AP2 domain. The latter proteins likely represent partial genes obtained through the transcriptome analysis. Similarly, all members of the RAV subfamily contained one AP2 domain and a B3 domain, except for three proteins that lacked the B3 domain and likely represent partial sequences. In addition, a subset of AP2 proteins contained YRG and YLG motifs.

### MeJA-induced changes in lignan biosynthesis

MeJA treatment of *I. indigotica* hairy roots cultures was employed to investigate changes in the biosynthesis of lignans (Figure 1A). The obtained results illustrated clear MeJA-inducibility of lignan biosynthesis both at the gene expression and metabolite accumulation level.

#### Transcript profiling demonstrates MeJA-induced changes of AP2/ERFs

**Figure 4:**
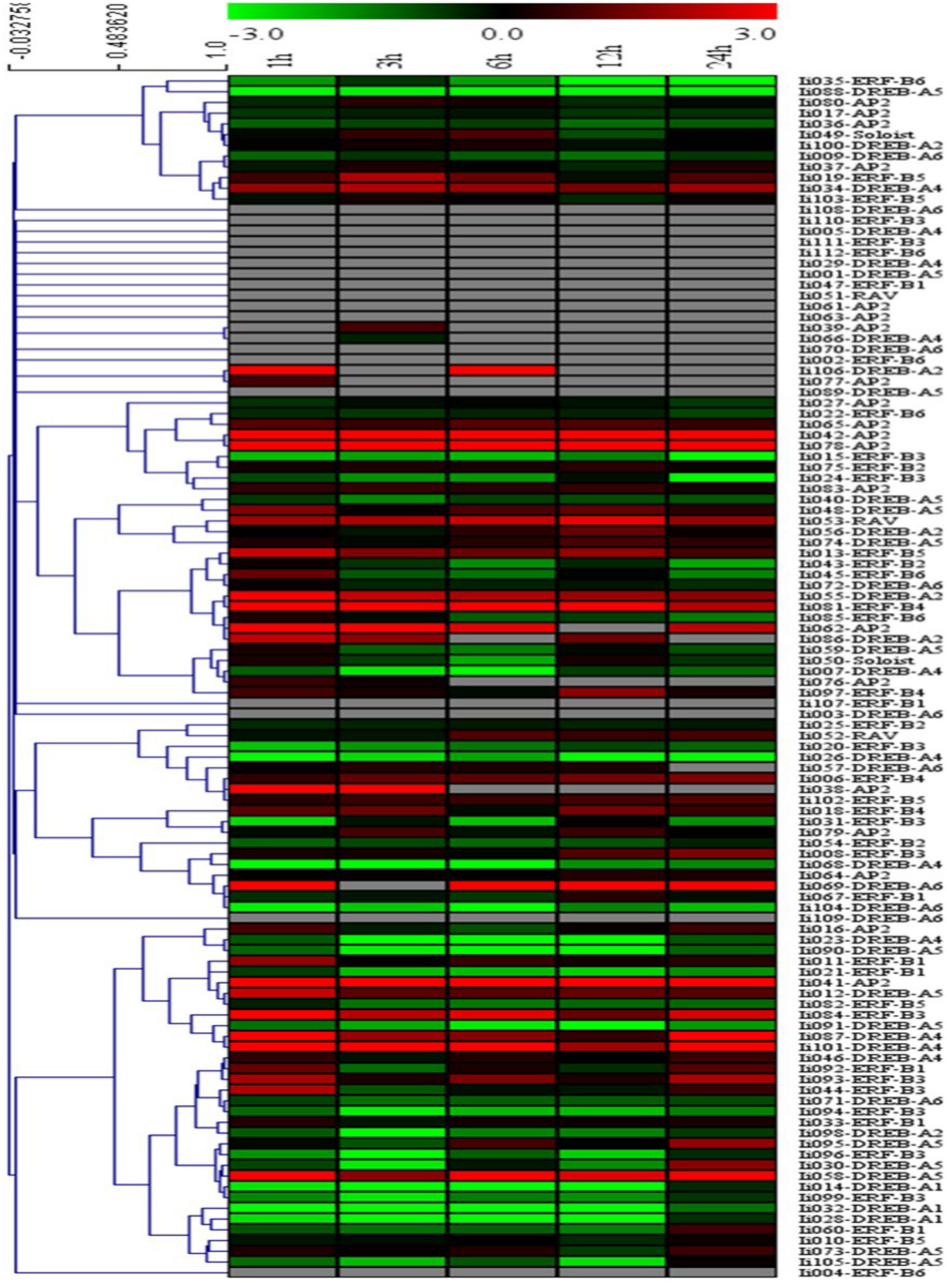
**Cluster analysis of the differentially expressed AP2/ERF genes identified in *I. indigotica***. Hairy roots of *I. indigotica* were treated with MeJA for 0, 1, 3, 6, 12, and 24 h and transcript abundance was measured via Illumina RNAseq analysis. The 0 h time point was used as control. Fold-change differences in transcript abundance are illustrated as heat map on a natural log scale (treatment/control). Samples with non-undetectable signals are depicted in gray.

Potential functions of the 112 putative AP2/ERFs were analyzed using Illumina RNAseq-based gene expression profiling in MeJA-treated *I. indigotica* hairy roots harvested 0, 1, 3, 6, 12, and 24 h post treatment and compared to non-treated samples. Changes in gene expression levels of AP2/ERFs inducible by MeJA are illustrated as a heat map (Figure 4). Of the 112 genes, 27 TFs were excluded from the study. Among the remaining genes, 13 TFs were up-regulated at 1, 3, 6, 12, and 24 h compared with 0 h, while 30 TFs were down-regulated. The remaining 42 TFs were up- or down-regulated at only individual time points. Notably, *Ii*04 and *Ii*078 were most highly up-regulated with 8.2- and 7.5-fold, respectively. Conversely, *Ii*014 and *Ii*068 were most highly down-regulated with 5.7- and 6.5-fold, respectively.

#### Verification of AP2/ERFs by qRT-PCR

To confirm the gene expression results obtained via RNAseq, 8 AP2/ERs were randomly chosen for additional qRT-PCR analysis. These genes comprised six up-regulated and two down-regulated genes upon MeJA treatment. As depicted in Figure S2, gene expression levels were comparable between RNA-seq- and qRT-PCR-derived results, supporting the reliability of gene expression levels obtained by Illumina transcriptome sequencing.

#### MeJA-induced changes of biosynthetic genes in the hairy root transcriptome

MeJA treatment of *I. indigotica* hairy root tissue significantly increased expression levels of genes with proposed functions in lignan biosynthesis. Transcript abundance of *IiPAL, Ii4CL, IiC4H, IiC3H, IiCAD, IiCCR, IiPLR*, and *IiDIR* were observed to be gradually induced and their sequences were listed in Table S7. Interestingly, *Ii4CL* and *IiPLR* were most abundant at 12 h post treatment, while other transcripts showed the highest abundance at 6 h. The levels of gene up-regulation varied from 2.7 (*IiPAL, IiPLR*), 3.0 (*Ii4CL*), 3.9 (*IiC4H*), 11.5 (*IiC3H*), 4.9 (*IiCAD*), 6.0 (*IiCCR*), and 6.1 (*IiDIR*) fold as compared to time point 0 h (Figure 1B).

#### MeJA-induced changes in the *I. indigotica* hairy root metabolite profile

Accumulation of four compounds (coniferin, lariciresinol, secoisolariciresinol, and pinoresinol) as key metabolites in the biosynthesis of lignans was enhanced by MeJA treatment, but at different levels. Coniferin showed the highest accumulation with a 2.1-fold increase after 24 h. The remaining metabolites showed highest abundance already after 12 h with 3.5-, 3.0- and 4.1-fold increases, respectively (Figure 1C).

### Integration of transcript and metabolite abundance analyses

A canonical correlation analysis using Pearson's correlation coefficient was performed to identify possibly correlations between the transcript profiles of the 112 *Ii*AP2/ERFs and eight biosynthetic genes, and the four investigated metabolites.

**Figure 5:**
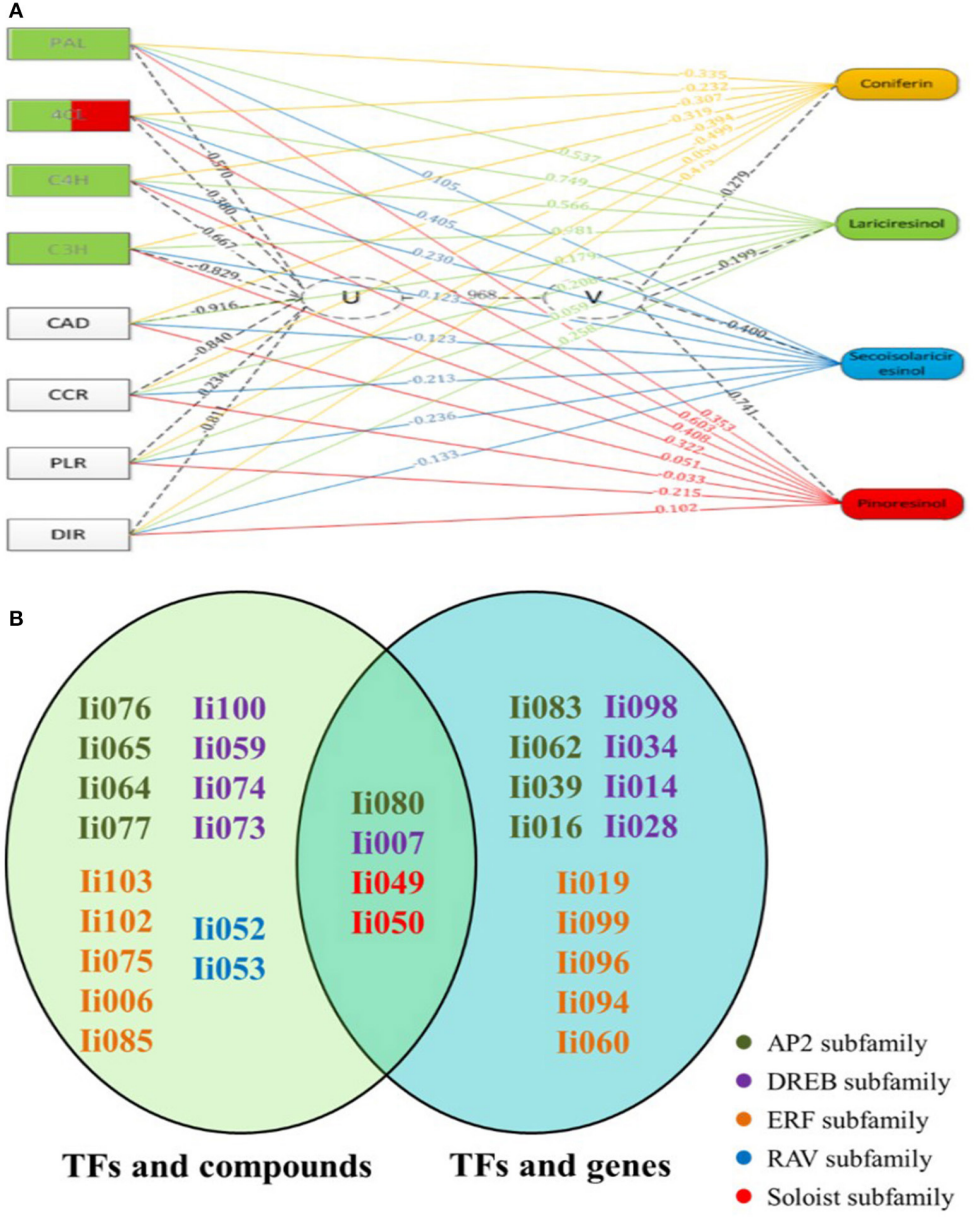
**Canonical correlation analysis using Pearson's correlation coefficient**. **(A)** Exemplary gene-to-metabolite network in MeJA-elicited *I. indigotica* hairy roots. Genes are depicted as squares on the left, and metabolites on the right. The canonical correlation coefficient between two canonical correlation variables (U and V) was 0.968. The correlation coefficient between raw variables (genes and metabolites) and canonical correlation variables (U and V), is illustrated as corresponding dotted lines. Number associated with lines represent the variable correlation coefficient, and the gene color illustrate the level of gene-to-metabolite correlation: edges depict variable correlation coefficients of >0.50 and blue represents a higher correlation to lariciresinol. **(B)** List of 19 AP2/ERFs with possible roles in regulating the accumulation of lignans. The left oval shows the result of canonical correlation analysis between AP2/ERFs and pathway metabolites, and the right oval shows the result of the analysis between AP2/ERFs and biosynthetic genes. Different families are color coded. Particularly, four common AP2/ERFs (*Ii*080, 007, 049, and 050) show high probability for functions regulating the biosynthesis of lignans.

As illustrated in Figure 5A, the first pair of canonical correlation variables (U and V) revealed a clear correlation between gene transcripts and target metabolites with a canonical correlation coefficient of 0.968. Detailed results of the complete correlation coefficients between raw variables (gene or metabolite) and canonical correlation variables (U or V) are listed in Tables S8, S9. To further investigate the gene-to-metabolite correlation structure, variable correlation coefficient cut-off values of 0.5 were applied. For example, the variable correlation coefficients showing the significance of correlations between *Ii4CL* transcript levels and accumulation of four metabolites (coniferin, lariciresinol, secoisolariciresinol and pinoresinol) were −0.23, 0.75, 0.41, and 0.60, respectively. These findings indicated that *Ii*4CL as a gene involved in the upstream biosynthetic pathway is correlated with lariciresinol and pinoresinol, but not or minimally correlated with coniferin and secoisolariciresinol.

Additional correlation analyses among TFs, biosynthetic genes and pathway intermediates that demonstrated a high average variable correlation coefficient were established in the same manner (Tables S8, S9). In summary, the performed study resulted in the below observations:

1. Lariciresinol showed a high correlation with *Ii4CL, IiC4H, IiC3H*, and *IiPAL*, which intriguingly almost all represent genes functioning in the up-stream pathway of phenylpropanoid metabolism. *IiC4L* showed the highest correlation with lariciresinol and pinoresinol.
2. As shown in Figure 5B, select members of the AP2 family (*Ii*076, 080, 065, 064, and 077) were highly correlated with all tested metabolites. In contrast, *Ii*080, 083, 062, 039, and 016 were significantly correlated with lignan biosynthetic genes. Similarly, select members of the DREB subfamily (*Ii*100, 059, 074, 007, 073) were also highly correlated with the tested metabolites, while other DREB proteins (*Ii*098, 007, 034, 014, 028) were correlated with lignan-biosynthetic genes, respectively. Among the ERF subfamily, *Ii*103, 102, 075, 006, 085 and *Ii*019, 099, 096, 094, 060 were highly correlated with the four compounds and biosynthetic genes, respectively. For *Ii*052 and 053 of the RAV family significant correlations were only observed with the four metabolites. *Ii*049 and 050 of the Soloist family again were highly correlated with both metabolites and biosynthetic genes. As for AP2/ERFs, *Ii*080 (AP2), *Ii*007 (DREB), *Ii*049, *Ii*050 (Soloist) showed simultaneous correlation with metabolites and biosynthetic genes. The three most significantly correlated TFs related to relevant pathway metabolites and biosynthetic genes are listed in Table S10. These correlations suggest a probable role of these TFs in lignan metabolism.
3. Based on these analyses, eight genes, namely *IiPAL, Ii4CL, IiC4H, IiC3H, Ii080, Ii007, Ii049*, and *Ii050*, are most likely to be involved in the biosynthesis of lariciresinol and provided promising targets for metabolic engineering approaches aimed at enhancing the yield of lariciresinol hairy root cultures.

### Metabolic engineering with *IiC3H* overexpression in *I. indigotica* hairy root cultures

**Figure 6:**
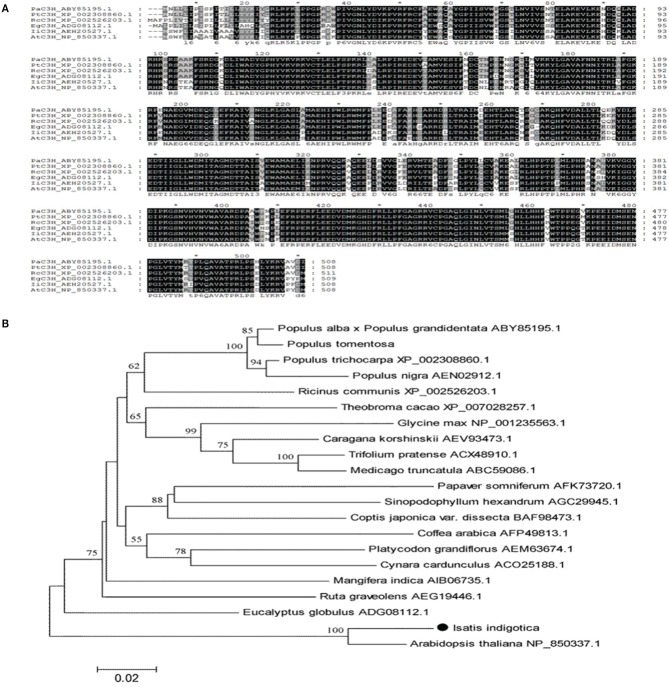
**Sequence and phylogenetic analysis of *Ii*C3H. (A)** Protein sequence alignment of *Ii*C3H with related proteins: *At*C3H in *A. thaliana* (NP_850337), *Pa*C3H in *Populus alba* (ABY85195), *Eg*C3H in *Eucalyptus globules* (ADG08112), *Pt*C3H in *Populus trichocarpa* (XP_002308860), and *Rc*C3H in *Ricinus communis* (XP_002526203); all containing a P450 domain. **(B)** Phylogenetic tree of C3H compared to known plant C3H enzymes.

Based on its proposed function in lignan biosynthesis, *IiC3H* was chosen for metabolic pathway engineering toward increased lariciresinol production in hairy root cultures. *IiC3H* (JF826963) represents a 1527 bp ORF encoding for a predicted 509 amino acid protein. *IiC3H* contains the characteristic P450 domains and BLAST analysis showed highest similarity to known coumarate 3-hydroxylases from other plant species, including *A. thaliana At*C3H (NP_850337)*, Populus alba Pa*C3H (ABY85195), *Eucalyptus globules Eg*C3H (ADG08112), *Populus trichocarpa Pt*C3H (XP_002308860), and *Ricinus communis Rc*C3H (XP_002526203) (Figure 6A).

A neighbor joining phylogenetic tree showed close relatedness of *Ii*C3H and *At*F3H, forming a separate cluster from other known plant C3H enzymes (Figure 6B). This suggests a possible functional relatedness of both proteins and highlights an expansive evolutionary diversification of the C3H family from a common P450 ancestor.

**Figure 7:**
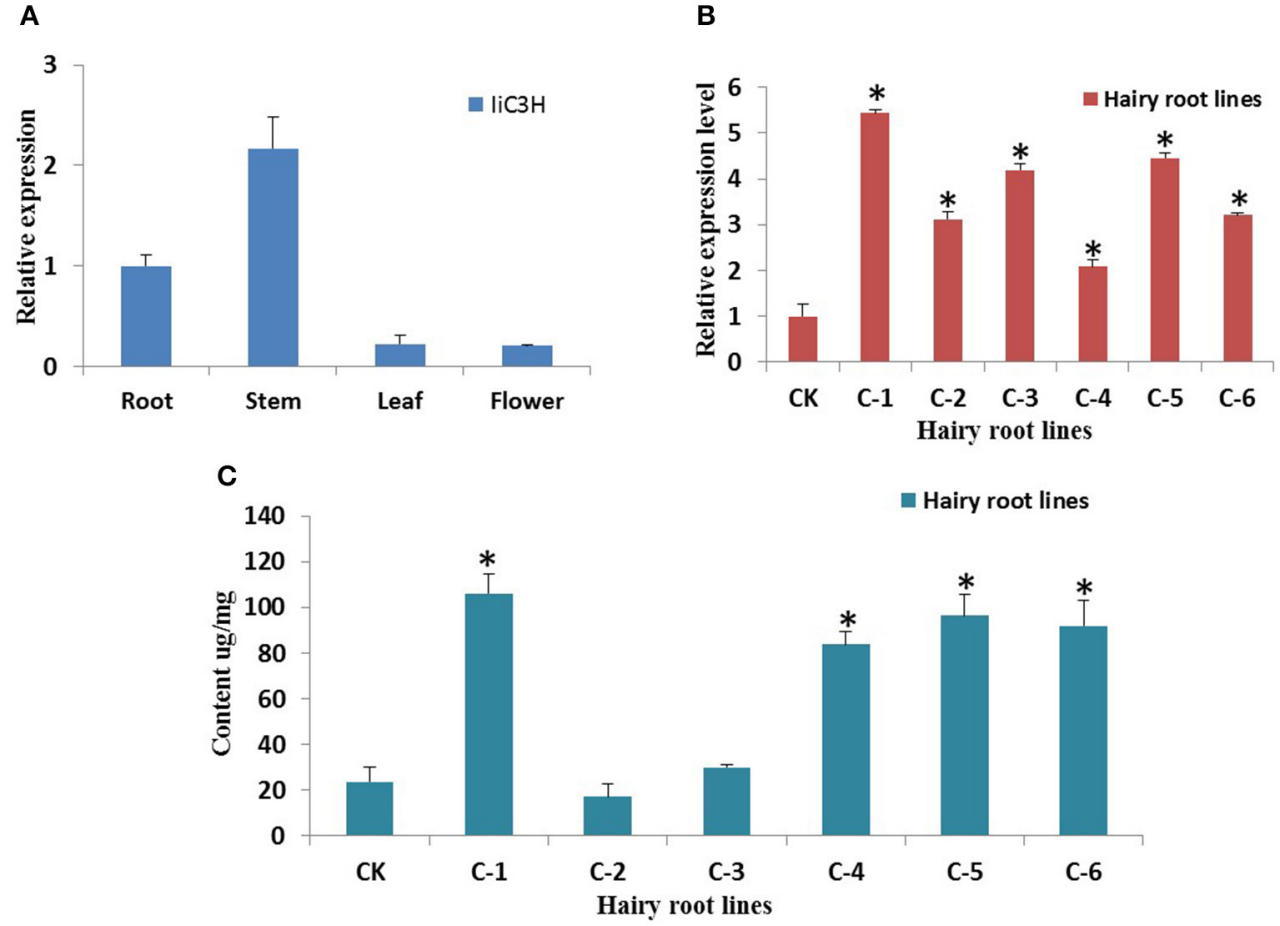
**Gene expression analysis of *IiC3H*. (A)** Tissue-specific transcript abundance of *IiC3H* in different organs of *I. indigotica*. The x-axis shows the different organs, and the y-axis represents the relative transcription levels of *IiC3H*. **(B)** Relative transcript abundance of *IiC3H* among different hairy root lines. The x-axis shows the different culture lines, and the y-axis represents the relative transcription levels of *IiC3H*, *n* = 3. **(C)** Accumulation of lariciresinol in different hairy root lines. The x-axis shows the different culture lines, and the y-axis represents the content of lariciresinol, *n* = 3. CK: negative control of wild type hairy roots; C1-C6: different monoclonal hairy root lines of *IiC3H* transformation. ^*^Significant difference between transgenic lines to CK, *p* < 0.01.

Tissue-specific gene expression analysis of *Ii*C3H in roots, stems, leaves, and flowers of *I. indigotica* using qRT-PCR revealed that *Ii*C3H was expressed predominantly in roots and stems (Figure 7A), which is consistent with previous studies demonstrating roots as the main organ for the synthesis and accumulation of lariciresinol (Chen et al., 2013).

**Figure 8:**
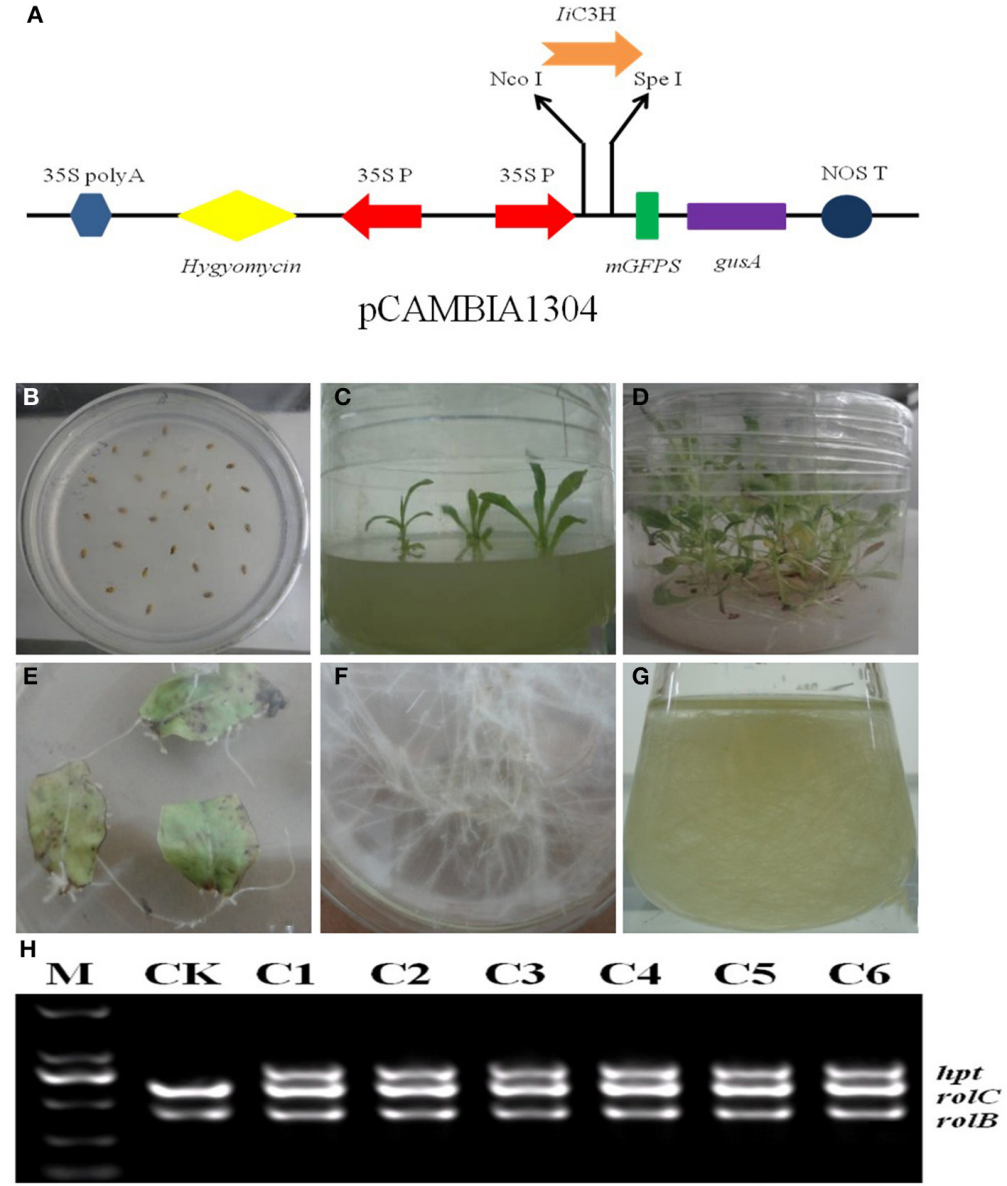
**Over-expression of *Ii*C3H in transgenic *I. indigotica* hairy root cultures. (A)** The full length ORF of *IiC3H* was inserted into the pCAMBIA1304 expression vector under control of the 35s promoter. **(B–D)** Germination stages of *I. indigotica* seeds. **(E–G)** Growth and production of hairy roots cultures. **(H)** PCR analysis of pCAMBIA1304-*IiC3H* in hairy roots. In *A. tumefaciens* strain C58C1, *rolB* and *rolC* represent DNA fragments of T-DNA in Ri, and *hpt* represents the hygromycin resistance gene of pCAMBIA1304, which indicated that pCAMBIA1304:*Ii*C3H had been successfully transformed into hairy roots. M, DL2000 maker; CK, negative control of wild type hairy roots; C1-C6, different monoclonal hairy root lines transformed with *Ii*C3H.

To increase lariciresinol biosynthesis engineered transgenic *I. indigotica* hairy root lines over-expressing *Ii*C3H were established. Here, the full length ORF of *Ii*C3H was inserted into the NcoI and SpeI sites of the pCAMBIA1304 expression vector (Figure 8A). Cultures of *I. indigotica* hairy roots were cultivated from seeds and transformed using *Agrobacterium tumefaciens* C58C1 (Figures 8B–G). Presence of the pCAMBIA1304-*Ii*C3H in transformed hairy roots was verified via PCR analysis (Figure 8H). In six hairy root lines (C1-C6), expression of *Ii*C3H was significantly up-regulated at 4.14-, 1.02-, 1.19-, 1.22-, 1.46-, and 2.21-fold compared to the control (CK), respectively (Figure 7B). At the same time, lariciresinol formation was increased by 4.45-, 0.72-, 1.25-, 3.5-, 4.1-, and 3.9-fold compared to the control in lines C1-C6, respectively. Using this approach, lariciresinol yields were increased from 23.8 to 96.4 mg·g^−1^ (Figure 7C), highlighting the important role of *Ii*C3H in the biosynthesis of lariciresinol and its utility for metabolic pathway engineering.

## Discussion

Through advanced whole genome sequencing model plants and high-throughput gene annotation function, systems biology and gene and/or metabolite network analyses have become increasingly powerful tools to elucidate the biosynthesis and regulation of plant secondary metabolism.

ERF proteins are known to play significant roles in signaling pathways in environmental interactions and the response to biotic and abiotic stress, as demonstrating through *in vivo* transgenic approaches in *A. thaliana* and many crop plants, such as rice (Giuntoli et al., 2014), tobacco (Zhu et al., 2014), and tomato (Klay et al., 2014). Yang and coworkers reported that *At*ERF073 (*AT*1G72360) modulated ethylene responses during hypoxia in *A. thaliana* (Yang et al., 2011). *Ii*054 showed high homology to *At*ERF073, suggesting a similar role in the response to hypoxia in *I. indig*otica. Furthermore, high homology of *Ii*109 with *At*ERF53 (*AT*2G20880), CaMV35S-controlled over-expression of which resulted in an unstable drought-tolerant phenotype in transgenic plants, may support a related functionality in drought tolerance (Cheng et al., 2012). The DREB family represented the largest AP2/ERF subfamily in *I. indig*otica. DREB proteins have frequently been used as viable candidates for enhancing crop abiotic stress tolerance (Gupta et al., 2014). Within this group, *Ii*028 was closely related to *At*DREB1A (*AT*4G25480) of *A. thaliana* involved the response to heat stress (Hong et al., 2009). Similarly, *At*DREB19 (*At*2g38340) and *Ii*086 are phylogenetically related and may have a similar functionality in enhancing tolerance to high salinity and drought stress (Krishnaswamy et al., 2011).

Members of the AP2 family have been associated with the shape and development of plant organs. For example, three *A. thaliana* mutants (*ap2-5, ap2-6*, and *ap2-7*) exhibited morphological changes of perianth organs (Kunst et al., 1989). Another member of the AP2 family, CRL5, impacted sepal abscission (Yan et al., 2012), plant height (*Ns*AP2) (Luo et al., 2012), and leaf shape (Jiang et al., 2012) in *Brassica napus*, water lily, and maize.

With respect to the RAV family, recent research on over-expressing *A. thaliana RAV1* suggested a role closely associated with leaf maturation and senescence (Woo et al., 2010). Similar roles related to plant senescence can be hypothesized for the members of the RAV family in *I. indigotica*, such as *Ii*051 and *Ii*052.

Although functional knowledge of the Soloist family is presently limited, the *A. thaliana* Soloist protein *At*4g13040 was shown to be a positive regulator of SA accumulation and basal defense against bacterial pathogens (Giri et al., 2014). Two homologous genes (*Ii*049 and *Ii*050) with possibly related activities were identified in *I. indigotica*.

**Figure 9:**
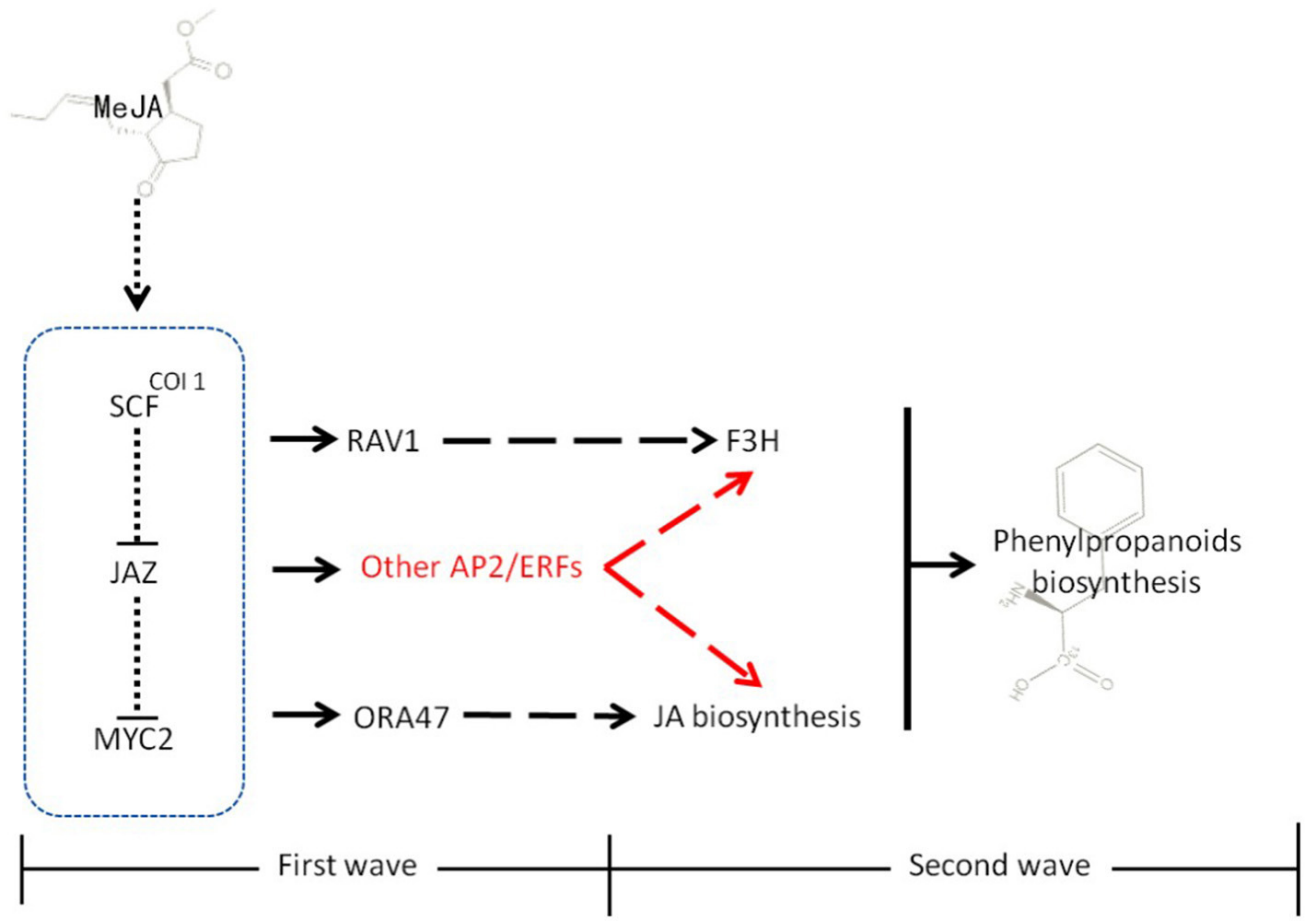
**Model of MeJA signal transduction in plants**. MeJA promotes the interaction of COI1 and SCF, leading to their degradation. In the first wave, these events induce the expression of a set of early responsive TFs that exert positive (MYC2) or negative (SZT/ZAT10, AZF2) control on JA biosynthesis. Some TFs, such as RAV1 directly interact with secondary metabolic genes. In second wave, the MeJA-mediated signaling cascade leads to transcriptional reprogramming of cellular metabolism toward increased phenylpropanoid and lignan biosynthesis and accumulation. In addition to RAV1 and ORA47, AP2/ERFs found in this study that might participate in the biosynthesis of lignans or generally phenylpropanoid metabolism are highlighted in red. Moreover, physical interactions (dotted lines), direct transcriptional regulation (solid lines), and incompletely characterized metabolic or signaling pathways (dashed lines) are highlighted.

As illustrated in Figure 9, studies in the model plant *A. thaliana* illustrated that MeJA-mediated stress responses (typically entailing modulation of different secondary metabolic pathways) proceed via two different but closely connected waves (Pauwels et al., 2008). In the first wave, MeJA induces the expression of select JA-biosynthetic genes. In the second, MeJA induces phenylpropanoid metabolism and other secondary metabolic pathways. Eight different groups of TFs, comprising members of the JAZ/TIFY, AP2/ERF, WRKY, bHLH, MYB, NAC, and C2H2 Zn finger families, were found to be enhanced after MeJA treatment in the first wave. AP2/ERF TFs, as one of major group of TFs together with MYB and bHLH proteins have important functions in biological processes such as stress response and control of secondary metabolism (Dietz et al., 2010; Pires and Dolan, 2010; Rushton et al., 2010). Therefore, it appeared plausible that TFs belonging to these groups would play key roles in stress-induced lignan biosynthesis in *I. indigotica*.

Recent studies showed that expression of PLOX3: fLUC (a key enzyme in JA biosynthesis) was increased more than three-fold when the transcriptional activators ORA47 (an AP2/ERF protein) and MYC2 (a bHLH protein) were over-produced. This over-expression was also accompanied by an induction of phenylpropanoid metabolism in the second wave. In contrast, expression of genes involved in transcriptional regulation was induced in the early wave. Both, ORA47 and MYC2 functioned as positive activators in JA formation, but the underlying mechanism has not been resolved. In wheat and rice, the RAV1 (an AP2/ERF TF) binding site was found in the promoter region of F3H (involved in flavonoid biosynthesis) (Himi et al., 2011). Therefore, AP2/ERFs are capable of coordinating phenylpropanoid metabolism directly through controlling gene expression of biosynthetic genes such as F3H, or indirectly through interaction with other signaling pathways, such as JA biosynthesis.

Based on these previous findings, we set out to investigate if putative AP2/ERFs in *I. indigotica*, such as ORA47 or RAV1, could play key roles in regulation gene expression and metabolite formation in the biosynthesis of lignans. To address this question, we performed a canonical correlation analyses of AP2/ERFs, lignan biosynthetic genes and pathway metabolites identified to be differentially regulated in *I. indigotica* hairy roots following MeJA treatment.

For this purpose, transcriptome and metabolite analyses were combined to discover key genes involved in lariciresinol biosynthesis in *I. indigotica* as an important medicinal plant. This study identified eight putative genes and *IiC3H* was chosen as an example. Over-expression of *IiC3H* was successfully employed to increase lariciresinol biosynthesis in transgenic hairy root cultures. In addition, four putative AP2/ERFs (*Ii*080, 007, 049, 050) were identified that show high probability to be involved in the regulation of lignan biosynthesis through interaction with pathway genes (similar to RAV1 in wheat) or via interaction with other signaling pathways (similar to ORA47 in *A. thaliana*).

## Author contributions

The study was conceived by RC, WC, and LZ. RC and QL collected the public dataset of *A. thaliana* and *B. rapa.* RC, JC, and RM contributed to data analysis, bioinformatics analysis, and manuscript preparation. SG, YX, and QL analyzed the accumulation of compounds through HPLC-MS/MS. RC, QL, and HT participated in planning of analyses and revising the manuscript. All authors have read and approved the final version of the manuscript.

### Conflict of interest statement

The authors declare that the research was conducted in the absence of any commercial or financial relationships that could be construed as a potential conflict of interest.

## Supplementary material

Figure S1 **AP2/ERF transcription factor comparisons across different species**.

Figure S2 **Quantitative real-time PCR analysis of eight select AP2/ERF transcripts**. Validation of the gene expression levels obtained via RNAseq was achieved by quantitative real-time PCR (qRT-PCR) analysis of eight randomly chosen AP2/ERF genes observed to be inducible by MeJA treatment, *n* = 3.

Table S1 **Oligonucleotides used in this study**.

Table S2 **Optimized MRM parameters for coniferin, lariciresinol, secoisolariciresinol, and pinoresinol**.

Table S3 **Putative homologous genes of 112 AP2/ERF sequences compared to *A. thaliana* and *B. rapa***.

Table S4 **Chemical and physical characteristics of 112 AP2/ERF proteins in *I. indigotica***.

Table S5 **Secondary structure prediction of AP2/ERF proteins**.

Table S6 **Prediction of subcellular localization**.

Table S7 **Sequences corresponding to the lariciresinol biosynthetic genes of *I. indigotica***.

Table S8 **Canonical correlation analysis**.

Table S9 **Analysis of correlation coefficient**.

Table S10 **Highest correlation among three compounds, three genes and five TFs**.
