# Supplementary material for: Gene-to-metabolite network for biosynthesis of lignans in MeJA-elicited Isatis indigotica hairy root cultures
Source: Front Plant Sci. 2015 Nov 3;6:952. doi: 10.3389/fpls.2015.00952 (PMC4630570; doi:10.3389/fpls.2015.00952)

Figure S1 AP2/ERF transcription factor comparisons among different species.

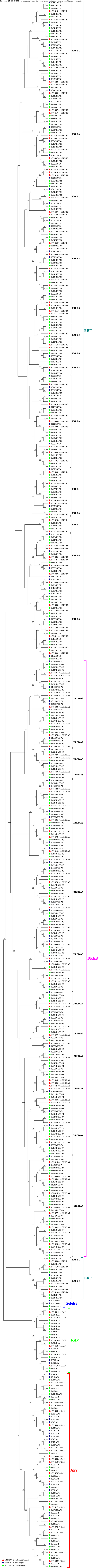

Supplement: Figure S1 — AP2/ERF transcription factor comparisons across different species. [file Figure S1.pdf › Revised supplementary data/Figure S1.pdf]
